# *Carex* *tianlinensis* and *C. leigongshanica*, Two New Species Related to *C. perakensis* from Southwest China, Based on Morphological and Molecular Evidence

**DOI:** 10.3390/plants15162481

**Published:** 2026-08-16

**Authors:** Zhaocen Lu, Yifei Lu, Zhenguo Xie, Ying Qin, Xiaofeng Jin

**Affiliations:** 1 School of Forestry and Biotechnology, Zhejiang A&F University, Hangzhou 311300, China; 2 Guangxi Key Laboratory of Plant Conservation and Restoration Ecology in Karst Terrain, Guangxi Institute of Botany, Guangxi Zhuang Autonomous Region and Chinese Academy of Sciences, Guilin 541006, China; 3 Administration of Guizhou Leigongshan National Nature Reserve, Leishan 557199, China

**Keywords:** Cyperaceae, morphology, phylogeny, new species, taxonomy, Southwest China

## Abstract

Two new species of *Carex* (Cyperaceae), *C. tianlinensis* and *C. leigongshanica*, are described and illustrated based on morphological and molecular evidence. Phylogenetic analyses based on two nuclear (ETS and ITS) and three plastid (*matK*, *trnL-F*, and *rpl32-trnL*^(UAG)^) DNA markers recovered *C. tianlinensis* and *C. leigongshanica* as two distinct, strongly supported monophyletic lineages within the *Indica* clade. The two new species form a well-supported sister pair that is in turn sister to *C. perakensis*. Morphologically, *C. tianlinensis* most closely resembles *C. perakensis*, but differs in having inflorescences arising near the apex of the culms, pistillate part of spikes sparsely 2–6-flowered, and style glabrous. *C. leigongshanica* is also morphologically similar to *C. perakensis*, but differs in having each inflorescence with 6–16 shorter spikes, spikes 0.7–1.5 cm long, sessile or subsessile, utricles shorter, 3–3.5 mm long, and leaf blades narrower, 3–5.5 mm wide. Detailed morphological descriptions, type designations, specimen citations, and information on habitat and phenology are provided for the two new species.

## 1. Introduction

The sedge family (Cyperaceae), comprising approximately 100 genera, is characterized by high species richness, a cosmopolitan distribution, marked disparities in lineage diversity, and a broad range of habitats [1]. Among these genera, *Carex* L. is the largest and most taxonomically challenging genus, distinguished by its unisexual flowers and highly reduced inflorescences, each bearing a single staminate or pistillate flower [1]. The pistillate flower is enclosed by a fully or partially fused prophyll, referred to as the utricle, which represents one of the most distinctive autoapomorphic innovations within the genus [2].

*Carex*, comprising more than 2000 species, is widely distributed across diverse ecosystems worldwide, including wetlands, forests, alpine meadows, and tundra regions [3,4]. The genus exhibits remarkable morphological diversity, particularly in utricle and nutlet characters, which have traditionally been used as key diagnostic traits for sectional delimitation and species identification [3,4,5]. These morphological characters are generally considered relatively stable within species and are thus of high taxonomic value. Nevertheless, widespread morphological convergence and continuous character variation within *Carex* have long posed significant challenges to species delimitation. Recent molecular phylogenetic studies have substantially reconstructed the infrageneric classification of *Carex*, recognizing six subgenera, 62 formally named Linnaean sections, and 49 informal groups [5].

### 1.1. Regional Diversity of Carex in China

In China, *Carex* comprises 632 species (including *Kobresia* Willd.), making it the second-largest genus of angiosperms after *Rhododendron* L. [6]. The species of *Carex* are widely distributed across a broad range of habitats, from lowland wetlands to alpine meadows and forest understories [7]. The highest species diversity is concentrated in Southwest China, particularly in Yunnan, Sichuan, Guangxi, and Guizhou, where complex topography, heterogeneous climatic conditions, and long-term environmental stability have promoted high levels of species richness and endemism [7]. These regions are widely recognized as biodiversity hotspots and refugia for many temperate and subtropical plant lineages [8]. However, many forest-dwelling sedges in these areas remain poorly studied, and their true diversity is likely underestimated owing to incomplete sampling and unresolved species complexes.

The region of Guangxi is highly diverse in *Carex* species. In recent years, intensified field surveys and comprehensive revisions of herbarium specimens have led to the reporting of many new species and newly recorded taxa, bringing the total number of *Carex* species in Guangxi to 129 [9,10], and making it the largest monocotyledoneous genus in the region. However, both systematic and taxonomic investigations have long been insufficient, resulting in uncertainties regarding species diversity, distribution patterns, and specimen identification. Species delimitation within the genus remains challenging due to extensive morphological variation, poorly understood interspecific boundaries, and overlap in reported diagnostic characters, which often hamper accurate identification. Moreover, several key areas, particularly the karst region of western Guangxi and other high-elevation montane habitats, remain inadequately explored, suggesting that the true diversity of *Carex* in Guangxi is likely still underestimated [9].

In the region of Guizhou, *Carex* is less diverse, and it is represented by 50 species and one variety, with a wide distribution across the province [11]. Given the region’s complex topography and diverse habitats, the actual diversity of *Carex* in Guizhou is likely underestimated, probably as a result of insufficient botanical surveys in many montane areas. Consequently, further field expeditions and detailed taxonomic studies are needed to more fully characterize the diversity of *Carex* in Guizhou.

### 1.2. The Carex Perakensis Group

Within the taxonomically complex *Carex*, *Carex perakensis* C.B.Clarke represents a widespread and morphologically variable species occurring in moist forests throughout southern China and Southeast Asia. Its inflorescence typically consists of 3–6 partial panicles, each bearing 3–7 androgynous spikes [7]. Historically, Kükenthal placed this species in subg. *Indocarex* Baill. based on inflorescence architecture [12]. Later, Raymond placed it in subg. *Carex*, emphasizing that its cladoprophylls are not utriculiform despite superficial similarities in inflorescence morphology [13]. This treatment was subsequently adopted in major floras, *viz. Flora Reipublicae Popularis Sinicae* [14] and *Flora of China* [7]. In the *Flora of Pan-Himalaya*, it is placed in the core *Carex* clade, within sect. *Decorae* (Kük.) Ohwi [15]. Current taxonomic frameworks place *C. perakensis* either in sect. *Decorae* based on morphological classifications or to the *Indica* clade based on phylogenetic evidence [5,7]. Both groups represent morphologically polymorphic and phylogenetically unresolved assemblages. Moreover, the substantial morphological variation observed in *C. perakensis* suggests that additional taxonomic diversity may remain unrecognized.

The taxonomic boundaries of *Carex perakensis* remain poorly understood. Its high morphological variability suggests that it may represent a species complex rather than a single evolutionary lineage. Previous studies have primarily focused on its position at the subgenus, section, or clade level, with little attention paid to the taxonomic implications of its morphological complexity for species delimitation. Moreover, comprehensive sampling and integrative phylogenetic analyses have been lacking for this group. As a result, potential cryptic diversity within *C. perakensis* and its allies has not been adequately explored.

During recent field investigations in Guangxi and Guizhou, Southwest China, together with the examination of extensive herbarium specimens, and two taxa morphologically similar to *C. perakensis* were discovered. Detailed morphological comparisons and molecular phylogenetic analyses based on nuclear and plastid DNA markers revealed that these taxa represent distinct evolutionary lineages. Herein, we describe these taxa as two new species, and name them *Carex tianlinensis* and *Carex leigongshanica* below.

## 2. Results

### 2.1. Comparison of Morphological Characters

A total of 256 herbarium specimens of *Carex perakensis* from South China, Southeast Asia, and South Asia, deposited in the herbaria (E, FJNU, HHBG, IBK, IBSC, K, KUN, KYO, LE, OKAY, P, PE, TI, and ZJFC), were available for measurement and comparison of morphological characters. Based on extensive examination of herbarium specimens and relevant taxonomic literature, *C. perakensis* was identified as the morphologically most similar species to both *C. tianlinensis* and *C. leigongshanica*. Moreover, molecular phylogenetic analyses (see below) recovered these three taxa as a well-supported clade, indicating that *C. perakensis* represents the most relevant species for diagnostic comparison. The diagnostic characters distinguishing *C. perakensis* from the two new species were consistently observed across all examined mature specimens. The morphological comparison of these three species is presented in Table 1.

### 2.2. Phylogenetic Relationships

**Figure 1:**
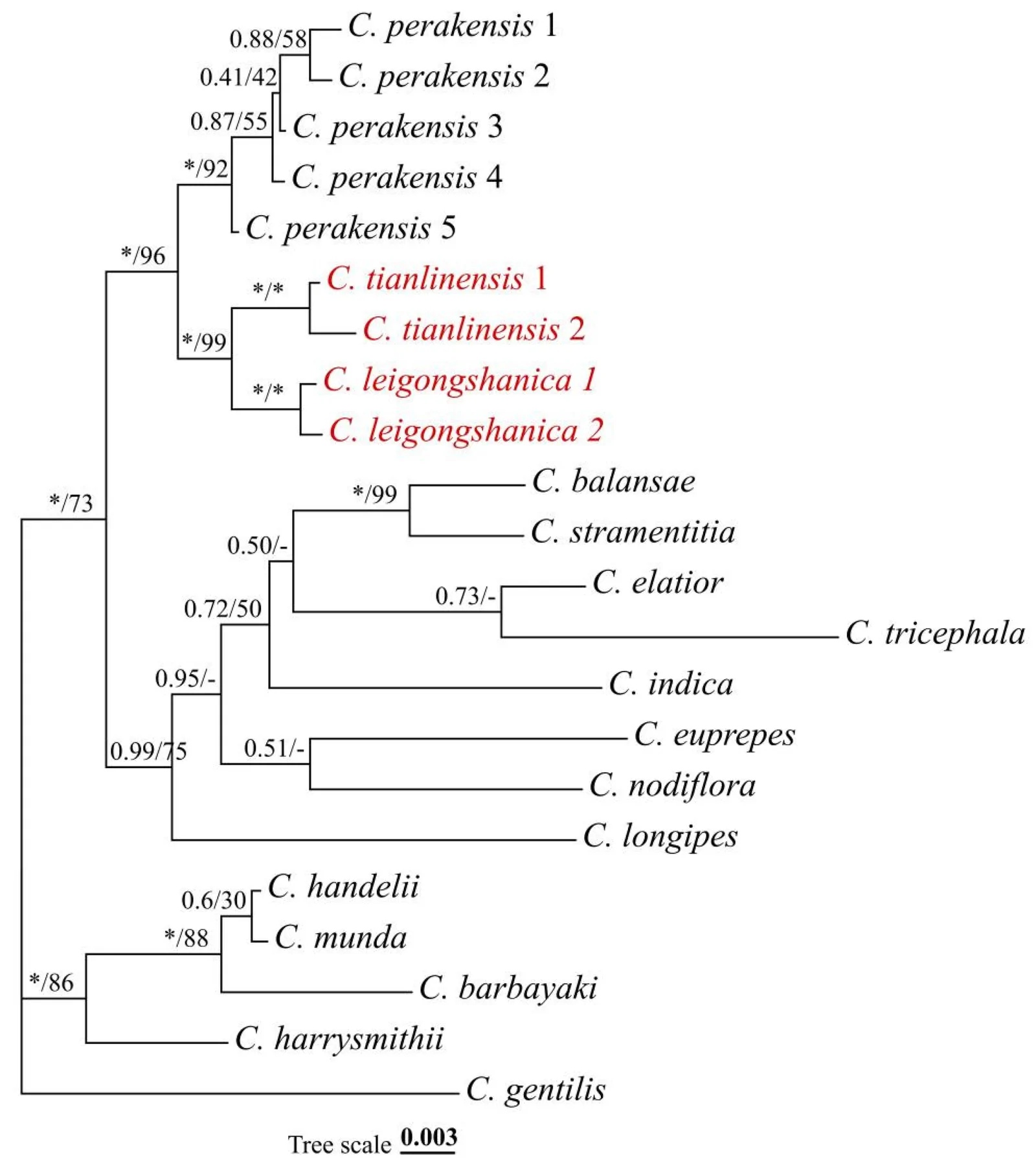
Bayesian inference (BI) and maximum likelihood (ML) phylogenetic tree of the *Indica* clade in Roalson et al. [5] derived from a combined dataset of ETS, ITS, *matK*, *rpl32-trnL*^(UAG)^, and *trnL-F*. Numbers above branches indicate Bayesian posterior probabilities (PP) and maximum likelihood bootstrap support values (BS, %), respectively. Asterisks (*) indicate nodes with maximum support (PP = 1.00 and BS = 100%). The new species are highlighted in red.

Following alignment and manual editing, the sequence lengths were 600 bp for ETS, 631 bp for ITS, 789 bp for *matK*, 844 bp for *rpl32-trnL*^(UAG)^, and 1029 bp for *trnL-F*. The best-fit nucleotide substitution models selected were GTR + G for ETS and ITS, GTR for *matK* and *rpl32-trnL*^(UAG)^, and GTR + I for *trnL-F*. The topologies resolved by Bayesian inference (BI) and maximum likelihood (ML) analyses were highly congruent, with no significant conflicts at strongly supported nodes (Figure 1). Both new species were nested within the *Indica* Clade proposed by Roalson et al. [5]. The two individuals of *Carex tianlinensis* and two of *C. leigongshanica* each formed strongly supported monophyletic clades (PP = 1.00, BS = 100%). These two clades together were recovered as sister lineages (PP = 1.00, BS = 99%), and together formed a well-supported clade sister to the sampled *C. perakensis* lineage (PP = 1.00, BS = 96%). The sampled of *C. perakensis* also formed a monophyletic clade (PP = 1.00, BS = 92%), although relationships among these samples were only weakly resolved.

## 3. Discussion

### 3.1. Phylogenetic Relationships and Taxonomic Implications

*Carex perakensis* has long been considered a morphologically variable species, and its systematic placement has changed repeatedly in previous classifications. Based on inflorescence architecture resembling a panicle structure, Kükenthal assigned it in sect. *Scabrellae* Kük. of subg. *Indocarex* [12]. Raymond subsequently assigned it to subg. *Carex*, emphasizing the cladoprophylls not utriculiform despite similarities in inflorescence architecture [13]. Raymond’s treatment of it was later adopted [7,14]. More recently, molecular phylogenetic studies have shown that many traditional subgenera and sections in *Carex* are not monophyletic [3,5]. In these phylogenetic classifications, *C. perakensis* has been variably placed within sect. *Decorae* or the *Indica* clade.

These different taxonomic treatments largely reflect the limited phylogenetic signal provided by several traditionally used morphological characters. Vegetative traits and overall inflorescence architecture among closely related species of the *Indica* clade show considerable similarity, and are prone to homoplasy, making species boundaries difficult to recognize based on morphology alone. Similar characters may evolve repeatedly in unrelated lineages, whereas closely related species may exhibit only subtle morphological differentiation [16]. Consequently, populations representing distinct evolutionary lineages have previously been treated as a single, morphologically variable species.

Our phylogenetic analyses recovered *C. tianlinensis* and *C. leigongshanica* as two strongly supported monophyletic lineages nested within the *Indica* clade, and resolved them as sister to *C. perakensis*. This placement is consistent with their overall morphological similarity, while the strong molecular support indicates that the observed morphological differences reflect lineage divergence rather than intraspecific variation. These findings suggest that other populations previously assigned to the broadly circumscribed *C. perakensis* may also represent multiple independently evolving lineages.

Although the concatenated plastid dataset contains a relatively high proportion of missing data, the phylogenetic results provide sufficient evidence to address the primary objective of this study, which was to evaluate the taxonomic placement of the two putative new species rather than to reconstruct a comprehensive phylogeny of the *Indica* clade. The two newly recognized species were consistently recovered as strongly supported monophyletic lineages in both BI and ML analyses, and their sister relationship was likewise strongly supported. Moreover, the molecular results are congruent with the observed morphological differentiation, providing robust evidence for recognizing the two new species. Nevertheless, we acknowledge that the high proportion of missing data may affect branch length estimation and support values at deeper nodes. Future studies incorporating broader taxon sampling and more complete molecular datasets will be valuable for evaluating the stability of relationships across the *Indica* clade.

The phylogenetic placement of the two newly recognized species suggests that *C. perakensis* may represent a species complex rather than a single morphologically variable species. Similar cases have been reported in other groups of *Carex*, where morphologically conserved taxa conceal substantial genetic divergence [17]. Our results therefore indicate that species diversity within the *Indica* clade may still be underestimated, and that additional taxonomic studies integrating broader geographic sampling and molecular evidence will be necessary to clarify species limits within this lineage.

### 3.2. Morphological Differentiation and Species Delimitation

Species delimitation in *Carex* has traditionally relied on reproductive morphology, particularly characters of the utricles, nutlets, pistillate glumes, and inflorescence, which are generally considered to be more stable and taxonomically informative than vegetative characters [2,18,19]. By contrast, vegetative characters such as plant size, leaf dimensions, and culm height often exhibit considerable phenotypic plasticity in response to environmental conditions and therefore show substantial overlap among closely related species [20].

In this study, the three species exhibit broadly similar vegetative morphology, but differ consistently in several reproductive traits, particularly spike number, spike size, pistillate flower number, utricle morphology, and style indumentum. These characters remained stable among all examined specimens, and correspond closely to the phylogenetic relationships recovered in this study, indicating that reproductive characters provide more reliable evidence for species delimitation than vegetative traits within the *C. perakensis* complex.

The coexistence of marked genetic differentiation and relatively limited morphological divergence suggests that morphological evolution within this group has occurred more slowly than lineage diversification. This morphological conservatism has been documented in *Carex* lineages [21,22], and may partly explain why widespread species have historically been regarded as highly variable taxa rather than as complexes of independently evolving lineages. In addition, convergent evolution of inflorescence architecture and vegetative morphology may further obscure species boundaries, making species delimitation based solely on morphology more difficult.

The morphological and phylogenetic evidence indicates that specimens previously identified as *C. perakensis* comprise multiple independent evolutionary lineages. The recognition of *C. tianlinensis* and *C. leigongshanica* as distinct species is supported by both stable morphological differences and phylogenetic evidence. These findings suggest that *C. perakensis* may represents a species complex rather than a single evolutionary lineage. This concordance between morphological and molecular evidence is consistent with previous studies showing that the integration of multiple lines of evidence provides a more reliable basis for species delimitation in morphologically conservative groups of *Carex* [20,21,22].

### 3.3. Species Diversity and Future Taxonomic Research

The discovery of *C. tianlinensis* and *C. leigongshanica* highlights that species diversity within the *Indica* clade remains incompletely documented, particularly in the montane regions of Southwest China. Together with previous studies, our findings suggest that morphologically conservative lineages may conceal additional taxonomic diversity, particularly where geographic exploration and molecular sampling remain limited. These results underscore the need for continued taxonomic investigation of the *Indica* clade.

Although our analyses provide strong morphological and phylogenetic evidence supporting the recognition of the two new species, molecular sampling for each new species remains limited, and taxon sampling within the *Indica* clade is still incomplete. Future studies with broader geographic and population-level sampling, together with phylogenomic data, will further refine species boundaries and evolutionary relationships within the *C. perakensis* complex.

## 4. Materials and Methods

### 4.1. Morphological Observation

Morphological observations of these two new species were conducted based on both living plants collected in the field and herbarium specimens. In total, 10 voucher gatherings of *Carex tianlinensis*, and 6 voucher gatherings of *C. leigongshanica* were examined. Particular attention was paid to vegetative and reproductive characters, including leaf size, inflorescence architecture, spike number and size, and the morphology and indumentum of the utricles and nutlets. Preliminary observation of these characters revealed that the two taxa were similar to *Carex perakensis*, a species widely distributed and frequently growing in moist places under forests in Southeast and East Asia. To evaluate their taxonomic status, herbarium specimens (including all type specimens) of *C. perakensis* and morphologically similar deposited species in E, FJNU, HHBG, IBK, IBSC, K, KUN, KYO, LE, OKAY, P, PE, TI, and ZJFC were critically examined and compared. Quantitative measurements are based on all available mature specimens examined. Throughout this study, utricle length was measured from the base of the utricle to the apex of the beak unless otherwise stated.

### 4.2. Taxon Sampling for Phylogenetic Analyses

To infer the phylogenetic positions of the two putative new species, we conducted phylogenetic analyses that included 22 samples representing 15 species of the *Indica* Clade, together with one representative of its sister clade selected as the outgroup, following the phylogenetic framework of Roalson et al. [5]. The *Indica* clade currently comprises 29 recognized species; however, comparable DNA sequences for the five selected markers are unavailable for many species. Our sampling includes 15 species, representing more than half of the currently recognized diversity of the clade, and encompassing the principal phylogenetic lineages recognized by previous studies. Importantly, the dataset includes *Carex perakensis*, the species morphologically most similar to the two putative new species, together with other closely related taxa, thereby providing an appropriate framework for evaluating their phylogenetic placement. Sequences of the two putative new species and three *C. perakensis* individuals were newly generated for this study, whereas the remaining sequences were downloaded from GenBank (Appendix A). For each taxon included in the phylogenetic analyses, all available DNA markers used in the concatenated dataset were derived from the same voucher specimen.

### 4.3. DNA Extraction, PCR Amplification, and Sequencing

Total genomic DNA was extracted from silica-dried leaf material using a modified CTAB method [23]. Five DNA regions, including two nuclear markers (ETS and ITS) and three plastid markers (*matK*, *rpl32-trnL*^(UAG)^, and *trnL-F*), were amplified and sequenced following the protocols described by Lu et al. [24]. Sequencing was performed on an ABI 3730 automated DNA sequencer (Applied Biosystems, Foster City, CA, USA).

### 4.4. Phylogenetic Analyses

Forward and reverse sequences were assembled using SeqMan in DNASTAR Lasergene v.8.1.3 [25], followed by alignment with MAFFT v.7, online version (https://mafft.cbrc.jp/alignment/server/, accessed on 10 June 2026) using the strategy of L-INS-i. Then the aligned sequences were trimmed and manually edited in MEGA v.7 [26]. The best nucleotide substitution model was evaluated for each gene region in jModelTest v.2.1.10 [27] using Akaike information criterion. Finally, five DNA regions were concatenated in PhyloSuite v.1.2.3 [28]. For the ML analysis, the concatenated dataset was analyzed under the GTR + I + G model in RAxML-HPC. Previous studies have shown that parameter-rich substitution models such as GTR + I + G generally produce phylogenetic estimates comparable to those obtained after exhaustive model selection [29]. Two strategies were used for phylogenetic analyses. A maximum likelihood (ML) tree was constructed using RAxML-HPC BlackBox v.8.2.12 [30] in the CIPRES Science Gateway (https://www.phylo.org/) under the GTR + I + G model with 1000 bootstrap replicates. Bayesian inference (BI) was conducted in MrBayes v.3.2.7a [31], two runs and four chains were carried out for 10^7^ generations with sampling of one tree every 1000 generations. A 50% majority-rule consensus tree was obtained after discarding the first 25% of all trees as burn-in. The phylogenetic trees were visualized in tvBOT [32].

## 5. Conclusions

### 5.1. Description of the New Species

**Figure 2:**
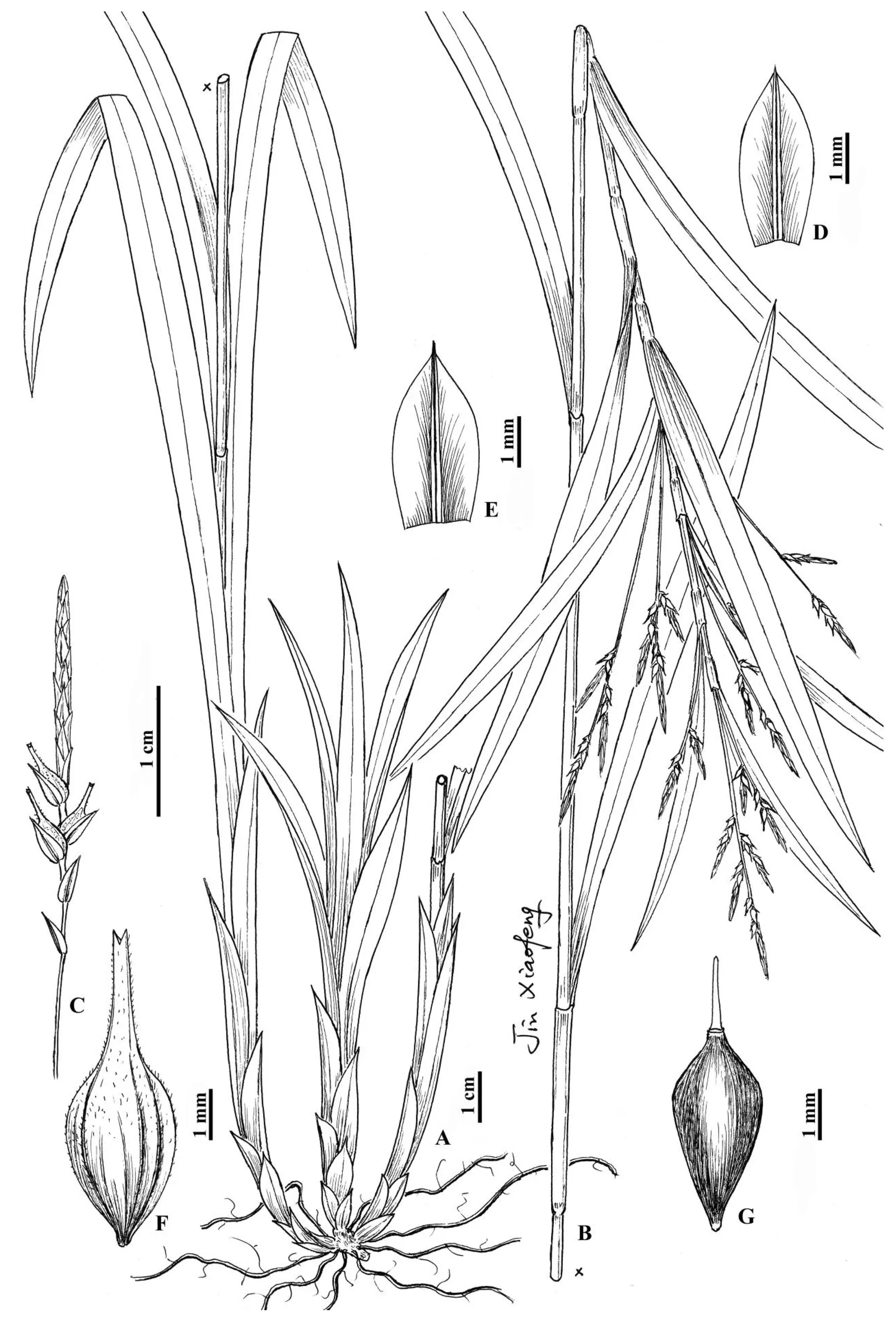
Line drawing of *Carex tianlinensis.* (**A**) and (**B**) habit; (**C**) spike; (**D**) male glume; (**E**) female glume; (**F**) utricle; (**G**) nutlet (drawn by Xiao-Feng Jin; based on the holotype: *Y. Qin* et al. *QYTL165* in IBK). “×” indicates the omitted portion of the culm.

**Figure 3:**
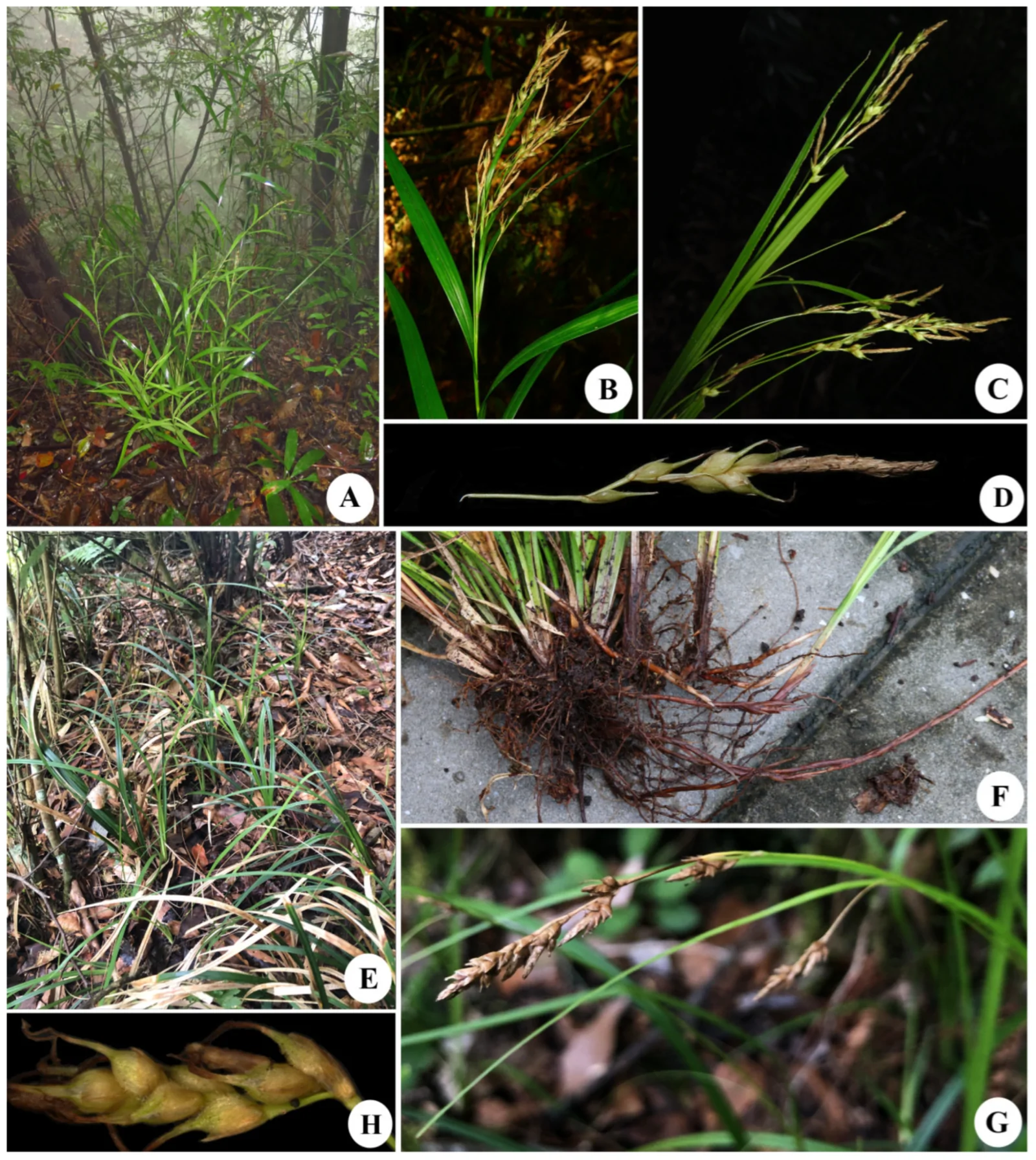
*Carex tianlinensis* (**A**–**D**). (**A**) habitat; (**B**,**C**) inflorescence; (**D**) spike. *Carex leigongshanica* (**E**–**H**). (**E**) habitat; (**F**) base of habit, showing rhizomes and stolons; (**G**) inflorescence; (**H**) spike.

*Carex tianlinensis* Z.C.Lu, Y.F.Lu & X.F.Jin sp. nov. (Figure 2 and Figure 3A–D)

Diagnosis: The new species is similar to *Carex perakensis*, but differs from the latter in having inflorescence arising from the upper part of the culm, pistillate part of spikes sparsely 2–6-flowered, and style glabrous.

Type: China. Guangxi: Tianlin, Mount. Cenwanglaoshan, Dalongping, on slope under open forest, 10 December 2019, *Y. Qin* et al. *QYTL165* (holotype: IBK!, barcode IBK00438089; isotypes: ZJFC!).

Description: Rhizomes woody, stiff. Culms central, loosely caespitose, 45–100 cm tall, lower part obtusely trigonous, upper part cylindrical, base with brown or dark brown sheaths, sometimes disintegrating into fibers. Leaves basal and cauline, slightly shorter than culms, cauline leaves with sheath at base; blades linear, 7–12 mm wide, subcoriaceous, apex acuminate, abaxially scabrous. Bracts leaf-like, nearly equal to inflorescence, rarely slightly longer, base long-sheathing, sheaths 3–12 mm long. Panicle compound, with 3–7 branched lateral inflorescences, 1–2 (3) lateral inflorescence arising from each bract sheath, with slender peduncles extending from bract sheath. Spikes 8–23, sessile or subsessile, androgynous, long-cylindrical, 8–25 mm long, staminate part 6–12 mm long, ca. 1.5 mm wide, densely flowered, pistillate part 4–10 mm long, 2–3.5 mm wide, sparsely 2–6-flowered. Staminate glumes narrowly obovate, pale yellow-brown, 3.5–4 mm long, apex acute, with yellow-white 3-veined costa. Pistillate glumes ovate, pale yellow, 3.5–4 mm long, apex acute, with pale brown 3-veined costa. Utricles obovoid or broadly obovoid (excluding beak), obtusely trigonous, pale yellow, 6–7 mm long (including beak), 1.5–2 mm wide, obliquely patent, distinctly laterally 2-veined and inconspicuously thinly veined, hispidulous along veins, apex gradually contracted into a 2.5–3 mm long beak, orifice obliquely truncate or 2-lobed with short teeth. Nutlets tightly enveloped, obovoid, trigonous, yellow-brown, ca. 4 mm long, ca. 2 mm wide, base shortly stipitate; style erect, base not thickened, glabrous; stigmas 3.

Etymology: The specific epithet ‘*tianlinensis*’ refers to the type locality of this new species, Tianlin County of Guangxi.

Phenology: Flowering and fruiting from late October to late December.

Distribution and habitat: *Carex tianlinensis* is known only from Cenwanglaoshan National Nature Reserve, Guangxi. It is sparsely distributed in the understory of slopes in evergreen and deciduous broad-leaved mixed forests, at an elevation of 700–1400 m.

Conservation assessment: The new species is currently known from a few collections in Cenwanglaoshan, Guangxi. However, no comprehensive field surveys have been conducted to determine its full distribution, population size, number of locations, and potential threats. Consequently, reliable estimates of its extent of occurrence (EOO) and area of occupancy (AOO) are not currently available. Following the IUCN Red List Categories and Criteria [33], the available information is insufficient, and the species is therefore provisionally assessed as Data Deficient (DD).

Additional specimens examined (paratypes): China. Guangxi, Tianlin, Mount. Cenwanglaoshan, from Dalongping Station to Houbeishan, on slope under dense thickets, 26 November 2019, *Y. Qin* et al. *QYTL026* (IBK!, barcodes IBK00438084, IBK00438085, IBK00438086), 27 November 2019, *Y. Qin* et al. *QYTL036* (IBK!, barcodes IBK00438087, IBK00438088); Dalongping Station, 106.389989° E, 24.398882° N, alt. 1391 m, *X. Y. Huang & J. Q. Dong CWA1763* (IBK!, barcodes IBK00450715, PE! barcode 02387041 and 02387158); Dalongping Station, Laoshan Village, 106.333518° E, 24.42617° N, alt. 1160 m, open forest along stream, *X. Y. Huang & P. Yang CWA2069* (IBK!, barcodes IBK00450716, PE! barcode 02387026 and 02387505); Lizhou Town, alt. 700 m, on slope under open forest, 31 October 2018, *Y. L. Jiang* et al. *LZC191* (IBK!, barcodes IBK00438090, IBK00438091); Langping, Taizi, 106.34561° E, 24.45410° N, alt. 1060 m, on slope under open forest, 19 November 2021, *C. L. Su* et al. *CWB1224* (IBK!, barcode IBK00450692, PE! barcode 02387159 and 02387639), near Tianwang Temple, 106.36026° E, 24.44378° N, alt. 1391 m, 27 November 2021, *C. L. Su* et al. CWB1535 (IBK! barcode IBK00450695, PE! barcode 02387526 and 02387527), the same locality, alt. 1400 m, under forest, 15 November 1988, *B. H. Chen & D. X. Zhang 176* (IBSC! barcode 0656386); without precise locality, alt. 1380 m, on slope, 28 November 1986, *Longtan Exped. 01215* (IBK!, barcodes IBK00211806).

Notes: *Carex tianlinensis* was first collected in 1986, and subsequently collected again in 1988, the two specimens were both collected from Cenwanglaoshan in Tianlin County of Guangxi, Southwest China, but were identified as *C. cryptostachys* Brongn. and *C. baccans* Nees respectively. During botanical diversity surveys conducted in the Cenwanglaoshan National Nature Reserve from 2018 to 2021, multiple additional specimens were collected, all of which had been identified as *C. hirtiutriculata* L.K.Dai in the China Virtual Herbarium (CVH). Recent comprehensive morphological and molecular phylogenetic studies have now confirmed it as a new species of *Carex*. Although these historical collections were previously misidentified, subsequent examination showed that *C. cryptostachys*, *C. baccans*, and *C. hirtiutriculata* are readily distinguishable from *C. tianlinensis* based on diagnostic reproductive characters. *Carex tianlinensis* is similar to *C. perakensis* in having spikes of similar length, utricles longer than glume, and style erect, but differs in having inflorescence arising from the upper part of the culm, pistillate part of spikes sparsely 2–6-flowered, and style glabrous.

Despite repeated collections within the reserve between 1986 and 2021, no systematic field survey of its wild populations has been carried out to date. As a result, its actual population size, distribution range, and potential habitats remain unclear. Given the high morphological similarity among species within the genus *Carex*, they are often overlooked or misidentified in field studies, which may lead to an underestimation of the diversity in regional assessments. We recommend implementing targeted surveys in similar habitats to better understand its conservation status and ecological distribution.

**Figure 4:**
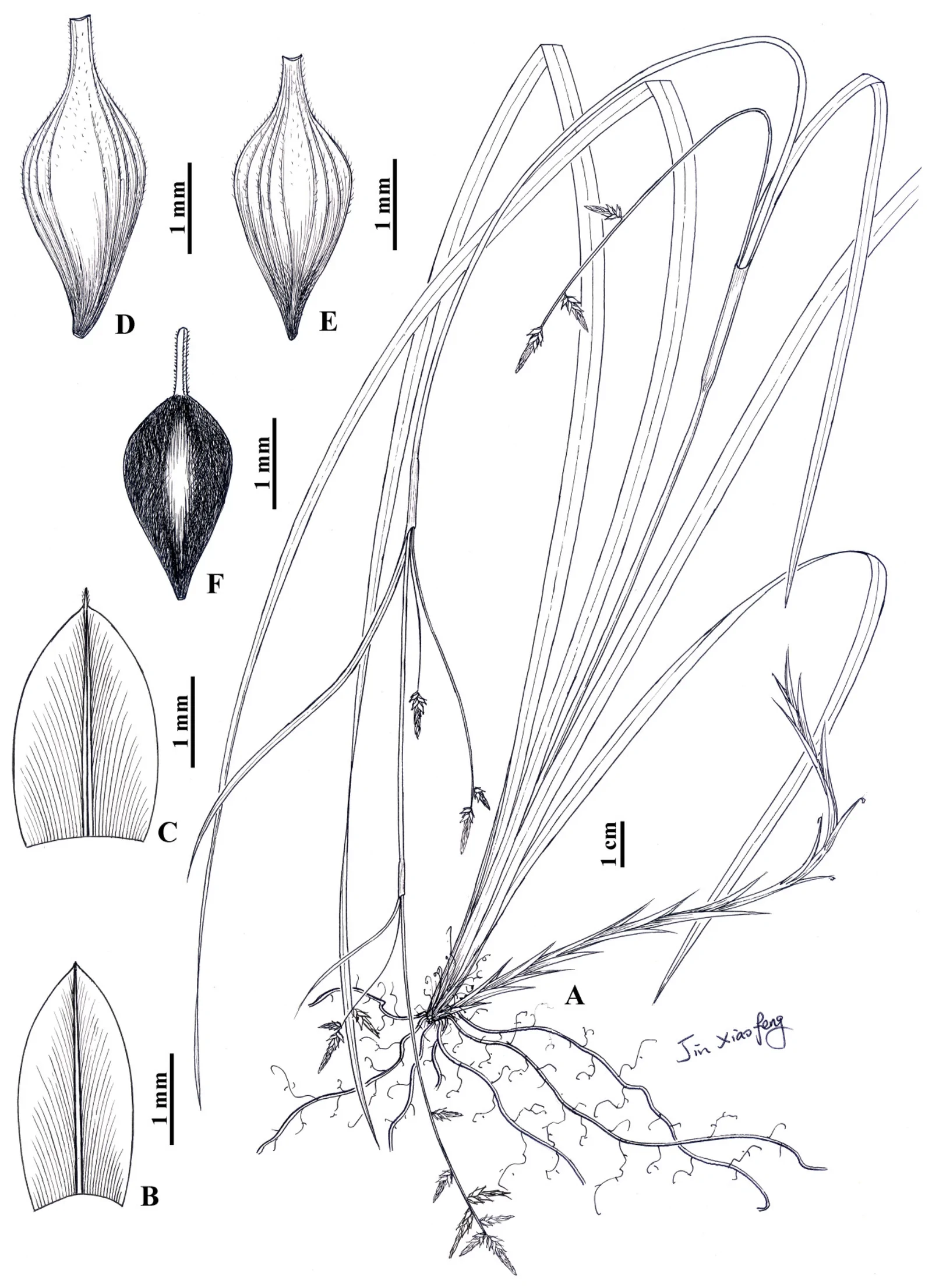
Line drawing of *Carex leigongshanica.* (**A**) habit; (**B**) male glume; (**C**) female glume; (**D**) and (**E**) utricles; (**F**) nutlet (drawn by Xiao-Feng Jin; based on the holotype: *X. F. Jin 4948* in ZJFC).

*Carex leigongshanica* X.F.Jin, Y.F.Lu & Z.G.Xie sp. nov. (Figure 3E–H and Figure 4)

Diagnosis: The new species is similar to *Carex perakensis*, but can be distinguished from the latter by its inflorescence with 6–16 spikes, spike 7–15 mm long, sessile or subsessile, utricles 3–3.5 mm long, and leaves 3–5.5 mm wide.

Type: China. Guizhou: Leishan County, Mount. Leigongshan, Xiannühu, 108.1978139° E, 26.3717889° N, alt. 1570 m, in grassland on slope under ever-green broad-leaved forest, 19 June 2021, *X. F. Jin 4948* (holotype: ZJFC!; isotypes: IBK!, PE!, ZM!).

Description: Rhizomes woody, stiff, long-stoloniferous. Culms central, loosely caespitose, 30–50 cm tall, obtusely trigonous, base with dark brown fibrous sheaths. Leaves basal, longer than or almost equal to culms; blades long-linear, 3–5.5 mm wide, leathery, apex acuminate, margin scabrous. Lower bracts leaf-like, longer than inflorescence, base long-sheathing, upper 3 or 4 bracts glume-like, apex scabrous long-awned. Panicle compound, with 3 or 4 branched lateral inflorescences, 1–2 lateral inflorescences arising from each bract sheath. Spikes 6–16, sessile or subsessile, androgynous, oblong-lanceolate, 7–15 mm long, staminate part 4–6 mm long, 1.5–2 mm wide, densely flowered, pistillate part 3–8 mm long, 3–4 mm wide, 3–8-flowered. Staminate glumes ovate, pale yellow-brown, 2.5–3.5 mm long, apex acute, with pale yellow 1-veined costa. Pistillate glumes broadly ovate, pale yellow-brown, 2.2–2.5 mm long, apex acute, with pale yellow 3-veined costa excurrent into a mucro. Utricles obovoid or broadly obovoid, yellow-green, obtusely trigonous, 3–3.5 mm long, 1.5–2 mm wide, obliquely patent, distinctly thinly veined, hispidulous along veins, apex abruptly contracted into a 0.5–0.7 mm long beak, orifice obliquely truncate or emarginate. Nutlets tightly enveloped, obovoid or broadly obovoid, trigonous, dark brown, 2–2.5 mm long, 1.2–1.5 mm wide, base shortly stipitate; style erect, barbate, base not thickened; stigmas 3.

Etymology: The specific epithet ‘*leigongshanica*’ refers to the type locality of this new species, Leigongshan, Leishan County of southeastern Guizhou Province.

Phenology: Flowering and fruiting from late June to late August.

Distribution and habitat: The new species is known only from Leigongshan National Nature Reserve, Guizhou. It occurs in montane forest understory, forest margins, and adjacent roadside habitats on slopes at elevations of ca. 1500 m.

Conservation assessment: *Carex leigongshanica* is currently known only from several collections made within Leigongshan National Nature Reserve, Guizhou. Although the species occurs in a protected area, its overall distribution, population size, habitat specificity, and potential occurrence outside the reserve remain poorly understood. Additional field surveys are needed to clarify its conservation status. We therefore provisionally assess the species as Data Deficient (DD) following the IUCN Red List Categories and Criteria [33].

Additional specimens examined (paratypes): China. Guizhou: Leishan County, Leigongshan Forest Farm, on slope under tickets, 28 August 1959, *S. Guizhou Exped.* (*Z.S.Zhang & Y.T.Chang*) *3716* (PE!); Leigongshan, 108.1978139° E, 26.3717889° N, roadside under forest, alt. 1532 m, 23 August 2019, *X. F. Jin* et al. *4524* (ZJFC)*, 4525* (ZJFC, ZM)*, 4526* (ZJFC); Xiannühu, grassland at forest margin, alt. 1564 m, 12 August 2023, *X. F. Jin & Y. F. Lu 5173* (ZJFC, ZM).

Notes: Leigongshan National Nature Reserve in southeastern Guizhou preserves well-developed mid-subtropical forests, and supports a rich flora with numerous endemic plant species [34]. Despite previous botanical investigations in this reserve, new taxa continue to be discovered, underscoring the importance of sustained floristic exploration in this understudied region.

*Carex leigongshanica* is currently known only from Leigongshan National Nature Reserve. Its discovery increases the known diversity of the *Indica* clade, and the surrounding mountains may reveal further taxonomic diversity. Nevertheless, further field investigations are required to clarify the species’ distribution, population status, and conservation significance.

### 5.2. Key to Carex tianlinensis, C. leigongshanica and C. perakensis

1a. Pistillate part of spikes sparsely 2–6-flowered; utricles 6–7 mm long, beak 2.5–3 mm long; style glabrous....................................................................................................*C. tianlinensis*
1b. Pistillate part of spikes sparsely 3–8-flowered or densely flowered; utricles 3–6 mm long, beak 0.5–2 mm long; style hispidulous or sparsely hispidulous...................................................2
2a. Leaf blades 3–5.5 mm wide; spikes 7–15 mm long; utricles 3–3.5 mm long, beak 0.5–0.7 mm long.......................................................................................................*C. leigongshanica*
2b. Leaf blades 4–12 mm wide; spikes 15–30 mm long; utricles 4.5–6 mm long, beak 1–2 mm long..................................................................................................................................*C. perakensis*

### 5.3. Integrative Approaches Needed to Resolve the Taxonomy of Carex perakensis Complex

Based on morphological and phylogenetic evidence, we describe two new species, *Carex tianlinensis* and *Carex leigongshanica*, from Southwest China. Both species are morphologically similar to *C. perakensis*, but can be distinguished by a combination of reproductive characters, including inflorescence architecture, spike number and length, and utricle length. Although vegetative characters show partial overlap among taxa, these reproductive features provide stable diagnostic differences consistent with species boundaries. Phylogenetic analyses based on two nuclear and three plastid DNA markers recover both new species as well-supported independent lineages, forming a clade sister to *C. perakensis*.

The two new species are currently known from localities in Guangxi and Guizhou, Southwest China, within subtropical broad-leaved forest habitats. At present, their distribution and wild populations remain unknown, further field surveys are needed to clarify their full geographic ranges and populations. The discovery of two new species further demonstrates that species diversity within the *C. perakensis* complex remains underestimated, and highlights the importance of integrative taxonomic approaches combining morphology and molecular phylogenetics in resolving species boundaries in *Carex*. Future phylogenetic and population-level studies are needed to better understand diversification processes and morphological evolution within this species complex.

## Figures and Tables

**Table 1:** Morphological comparison among *Carex tianlinensis*, *C. leigongshanica*, and *C. perakensis*.

Character	*C. tianlinensis*	*C. leigongshanica*	*C. perakensis*
Culms	45–100 cm tall, lower part obtusely trigonous, upper part cylindrical	30–50 cm tall, obtusely trigonous	30–120 cm tall, trigonous
Leaf blades	7–12 mm wide	3–5.5 mm wide	4–12 mm wide
Bracts	leaf-like	lower ones leaf-like, upper 3–4 glume-like	lower ones leaf-like, upper ones glume-like
Panicles	with 3–7 branched lateral inflorescences, 1–2 per sheath	with 3–4 branched lateral inflorescences, 1–2 per sheath	with 3–6 partial panicles, the lowermost one single or binate
Spikes	8–23, sessile or subsessile, long-cylindrical, 8–25 mm long	6–16, sessile or subsessile, oblong-lanceolate, 7–15 mm long	3–7 per partial panicle, sessile, lanceolate or oblong-lanceolate, 15–30 mm long
Pistillate part of spikes	sparsely 2–6-flowered	sparsely 3–8-flowered	densely flowered
Pistillate glumes	ovate, 3.5–4 mm long, with pale brown 3-veined costa	broadly ovate, 2.2–2.5 mm long, with pale yellow 3-veined costa excurrent into a mucro	broadly ovate to oblong, 2.2–4.5 mm long, with 1-veined costa excurrent into a short awn
Utricles	obovoid or broadly obovoid, 6–7 mm long; beak 2.5–3 mm long	obovoid or broadly obovoid, 3–3.5 mm long; beak 0.5–0.7 mm long	obovate-elliptic or ovate-fusiform, 4.5–6 mm long; beak 1–2 mm long
Nutlets	obovoid, ca. 4 mm long, yellow-brown	obovoid or broadly obovoid, 2–2.5 mm long, dark brown	obovoid-ellipsoid, 2.5–3 mm long, dark brown
Styles	glabrous	hispidulous	sparsely hispidulous

## Data Availability

The newly generated sequences have been deposited in the National Center for Biotechnology Information (NCBI) GenBank database. All data supporting the findings of this study are included in the main text.

## Appendix A

The GenBank accession numbers of sequences used in the phylogenetic analyses (ETS, ITS, *matK*, *rpl32-trnL*^(UAG)^, and *trnL-F*) are provided. Voucher specimens are provided for species from which new sequences were generated. An en dash (–) indicates that no sequence was available for that marker.

*Carex balansae*: MN760447, MN762644, MN763034, –, –; *C. barbayaki*: –, –, MN763291, –, –; *C. elatior*: MN760777, –, MN763536, –, –; *C. euprepes*: MN759802, MN762645, MN763038, –, –; *C. gentilis*: OR913366, OR689451, OR866332, OR913416, OR913300; *C. handelii*: MN760019, MN762076, MN763514, –, –; *C. harrysmithii*: MT920738, MT920770, MT920803, MT920842, MT920878; *C. indica*: MN760793, MN761807, MN763237, –, –; *C. leigongshanica* 1 (China, Guizhou, Leishan, Voucher: *X.F.Jin* et al. *4524* (ZJFC)): PZ530854, PZ527501, PZ530861, PZ530868, PZ530873; *C. leigongshanica* 2 (China, Guizhou, Leishan, Voucher: *X.F.Jin* et al. *4525* (ZJFC)): PZ530855, PZ527502, PZ530862, PZ530869, PZ530874; *C. longipes*: MH329378, MH348248, MH496704, –, –; *C. munda*: MN760017, –, MN763376, –, –; *C. nodiflora*: –, MN762469, –, –, –; *C. perakensis* 1: MN759992, MN762592, MN763036, –, –; *C. perakensis* 2 (China, Guangxi, Debao, Voucher: *W.B.Xu* et al. *15135* (ZJFC)): PZ530859, PZ527506, PZ530866, PZ530871, PZ530878; *C. perakensis* 3 (China, Guangxi, Tianlin, Voucher: *P.Yang* et al. *LZC262* (ZJFC)): PZ530860, PZ527507, PZ530867, PZ530872, PZ530879; *C. perakensis* 4: MN760771, MN762594, MN763037, –, –; *C. perakensis* 5 (China, Guangxi, Tianlin, Voucher: *Y.Qin 42* (ZJFC)): PZ530856, PZ527503, PZ530863, PZ530870, PZ530875; *C. stramentitia*: MN760153, MN762596, MN763032, –, –; *C. tianlinensis* 1 (China, Guangxi, Tianlin, Voucher: *Y. Qin* et al. *QYTL165* (ZJFC)): PZ530858, PZ527505, PZ530865, –, PZ530877; *C. tianlinensis* 2 (China, Guangxi, Tianlin, Voucher: *Y. Qin* et al. *QYTL036* (IBK)): PZ530857, PZ527504, PZ530864, –, PZ530876; *C. tricephala*: –, MN762001, –, –, –.
